# Supplementary material for: Effectiveness and Safety of Ayurvedic Medicines in Type 2 Diabetes Mellitus Management: A Systematic Review and Meta-Analysis
Source: Front Pharmacol. 2022 Jun 8;13:821810. doi: 10.3389/fphar.2022.821810 (PMC9213670; doi:10.3389/fphar.2022.821810)
Supplement: Supplementary file 1 [file DataSheet1.zip › Supplementary Material/Forest and Funnel Plots/Nigella sativa/TG.pdf]

Nigella sativa - TG

Study	Intervention N	Intervention Mean	Intervention SD	Comparator N	Comparator Mean	Comparator SD	Mean Diff. with 95% CI	Weight (%)
Najmi 2012	40	150.39	38.92	40	155.01	16.97	-4.62 [-17.78, 8.54]	44.75
Hosseini 2013	35	174.40	55.80	35	191.40	57.60	-17.00 [-43.57, 9.57]	11.08
Hadi 2015	23	139.00	62.70	20	159.00	55.80	-20.00 [-55.72, 15.72]	6.14
Heshmati 2015	36	214.90	70.50	36	244.40	59.80	-29.50 [-59.70, 0.70]	8.58
Kooshki 2020	27	165.90	67.20	23	180.70	62.60	-14.80 [-51.02, 21.42]	5.97
Jangjo-Borazjani 2021	20	102.80	22.87	20	118.00	34.74	-15.20 [-33.43, 3.03]	23.47
**Overall**							-12.16 [-21.02, -3.30]	

Heterogeneity: $\tau^2 = 0.59$, $I^2 = 0.41\%$, $H^2 = 1.00$

Test of $\theta_i = \theta_j$: $Q(5) = 2.97$, $p = 0.70$

Test of $\theta = 0$: $z = -2.69$, $p = 0.01$

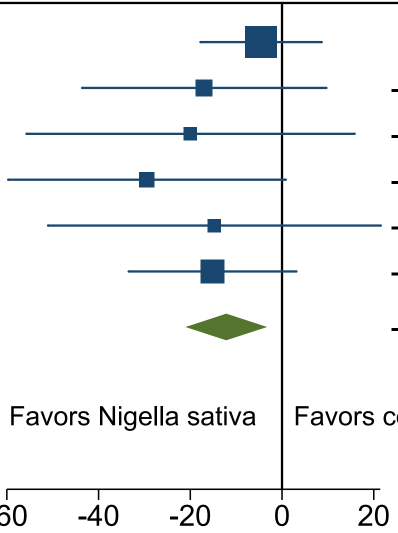

Random-effects REML model
